# A change of PD-1/PD-L1 expression on peripheral T cell subsets correlates with the different stages of Alzheimer's Disease

**DOI:** 10.1186/s13578-022-00897-1

**Published:** 2022-09-30

**Authors:** Ching-Tse Wu, Cheng-I Chu, Feng-Yu Wang, Hui-Yu Yang, Wei-Sung Tseng, Chuang-Rung Chang, Chien-Chung Chang

**Affiliations:** 1 Institute of Biotechnology, National Tsing Hua University, Hsin-Chu, Taiwan; 2 Department of Neurology, National Taiwan University Hospital Hsin-Chu Branch, Hsin-Chu, Taiwan; 3 Institute of Biochemical Sciences, National Taiwan University, Taipei, Taiwan; 4 Institute of Biotechnology, National Taiwan University, Taipei, Taiwan; 5 Department of Medical Science, National Tsing Hua University, Hsin-Chu, Taiwan; 6 Department of Life Science, National Tsing Hua University, Hsin-Chu, Taiwan; 7 Institute of Cellular and Molecular Biology, National Tsing Hua University, Hsin-Chu, Taiwan

**Keywords:** Alzheimer’s disease, Immune checkpoint, PD-1, PD-L1, Immunotherapy, Cognitive impairment

## Abstract

**Background:**

Immune checkpoints are a set of costimulatory and inhibitory molecules that maintain self-tolerance and regulate immune homeostasis. The expression of immune checkpoints on T cells in malignancy, chronic inflammation, and neurodegenerative diseases has gained increasing attention.

**Results:**

To characterize immune checkpoints in neurodegenerative diseases, we aimed to examine the expression of the immune checkpoint PD-1/PD-L1 in peripheral T cells in different Alzheimer’s disease (AD) patients. To achieve this aim, sixteen AD patients and sixteen age-matched healthy volunteers were enrolled to analyze their CD3^+^ T cells, CD3^+^CD56^+^ (neural cell adhesion molecule, NCAM) T cells, CD4^+^/CD8^+^ T cells, and CD4^+^/CD8^+^CD25^+^ (interleukin-2 receptor alpha, IL-2RA) T cells in this study. The expression of PD-1 on T cells was similar between the AD patients and healthy volunteers, but increased expression of PD-L1 on CD3^+^CD56^+^ T cells (natural killer T cells, NKT-like), CD4^+^ T cells (helper T cells, Th), CD4^+^CD25^+^ T cells, and CD8^+^ T cells (cytotoxic T lymphocytes, CTL) was detected in the AD patients. In addition, we found negative correlations between the AD patients’ cognitive performance and both CD8^+^ T cells and CD8^+^CD25^+^ T cells. To identify CD8^+^ T-cell phenotypic and functional characteristic differences between the healthy volunteers and AD patients in different stages, a machine learning algorithm, t-distributed stochastic neighbor embedding (t-SNE), was implemented. Using t-SNE enabled the above high-dimensional data to be visualized and better analyzed. The t-SNE analysis demonstrated that the cellular sizes and densities of PD-1/PD-L1 on CD8^+^ T cells differed among the healthy, mild AD, and moderate AD subjects.

**Conclusions:**

Our results suggest that changes in PD-1/PD-L1-expressing T cells in AD patients’ peripheral blood could be a potential biomarker for monitoring disease and shed light on the AD disease mechanism. Moreover, these findings indicate that PD-1/PD-L1 blockade treatment could be a novel choice to slow AD disease deterioration.

**Supplementary Information:**

The online version contains supplementary material available at 10.1186/s13578-022-00897-1.

## Background

Alzheimer’s disease (AD) is characterized by cerebral lesions, such as amyloid plaque and phosphorylated tau. Both lesions exhibit a specific cellular and regional vulnerability pattern. Regarding the cause of the cerebral pathological changes, recent studies focusing on the postinfectious model [1], mitochondrial dysfunction [2], and vascular risk factors [3] revealed the complexity of AD pathogenesis. Over the past half-century, several studies have indicated that inflammatory molecules are either uniquely presented or significantly elevated in AD patients’ brains. These molecules include interleukin (IL)-1 [4, 5], tumor necrosis factor-α (TNF-α) [6], and C-reactive protein (CRP) [7]. The indication of chronic brain inflammation is thought to be linked to neuronal death in AD. Based on these findings, anti-inflammatory agents, such as nonsteroidal anti-inflammatory drugs (NSAIDs), were proposed for the treatment of AD patients [8, 9]; however, no current anti-inflammatory agents are effective in preventing AD progression.

Previous research has demonstrated that proinflammatory T cells migrate across the compromised blood‒brain barrier (BBB) and induce neuroinflammation in neurodegenerative diseases, such as multiple sclerosis, Parkinson’s disease, and AD [10]. Inflammatory CD4^+^ T cells, including type 1 helper T (Th1), Th17, GM-CSF-producing CD4^+^ T cells, and γδ T cells, may recognize central nervous system (CNS) antigens and contribute to neuroinflammation and the progression of neurodegenerative diseases [11]. In addition to the cerebral infiltration of inflammatory T cells, the altered distribution of lymphocytes [12] and the increased suppressive activity of CD4^+^CD25^+^Foxp3^+^ regulatory T cells (Tregs) were discovered in AD patients’ peripheral blood [13]. In patients with mild AD, the proportion of activated CD8^+^ T cells, which might be related to AD-typical neuropsychological deficits, is significantly increased in peripheral blood and cerebrospinal fluid (CSF) [14]. Moreover, CD4^+^ peripheral lymphocytes exhibit mitochondrial dysfunction in early-stage AD patients [15]. In summary, alterations in lymphocyte populations and activities in the peripheral blood and CSF of AD patients have been widely reported.

Adaptive immunity is induced by lymphocyte-mediated antigen recognition. In AD patients, amyloid-β (Aβ) [16], herpes simplex virus, and Epstein‒Barr virus (EBV) [17, 18] are thought to be antigens that initiate adaptive immune responses. Regarding Aβ, no neuroinflammation was observed despite the peripheral activation of functional Aβ-specific CD8^+^ T cells and enhanced CD8^+^ T-cell infiltration in the brain in APPPS1 mice [19]. Furthermore, Gate et al. identified clonally expanded EBV-specific CD8^+^ T cells in AD patients' CSF, linking the viral immune response and AD [20]. Interestingly, EBV infects approximately 90% of adults worldwide [21]. The critical question is why some populations have a higher prevalence of AD and what are their common characteristics. A potential reason might be differences in the regulation of immune homeostasis.

The immune checkpoint, a set of costimulatory and inhibitory molecules, maintains self-tolerance and regulates immune homeostasis. Two well-studied immune checkpoints, programmed death-1 (PD-1) and its ligand (PD-L1), have been delineated as immune regulators in adaptive immunity. PD-1 is expressed by T cells [22], B cells [23], myeloid cells, thymocytes [24], and natural killer (NK) cells [25], and PD-L1 is expressed by antigen-presenting cells (APCs) [26] and macrophages [27]. The interaction between the extracellular domain of PD-1 and PD-L1 induces conformational changes in the PD-1 cytoplasmic domain and attenuates T-cell-activating signals [28]. To date, therapeutic monoclonal antibodies targeting the PD-1/PD-L1 pathway have been used for the treatment of patients with late-stage cancer [29, 30]. Similar to its essential role in maintaining immune homeostasis, the engagement of the PD-1/PD-L1 pathway in AD has also been revealed. Two separate studies demonstrated that PD-1/PD-L1 blockade increases cerebral amyloid plaque clearance and improves cognitive performance in different murine models [31, 32]. Noticeably, previous studies demonstrated the difference in the amount of peripheral PD-1^+^CD4^+^ T cells between AD patients and healthy volunteers. These results indicate the potential role of the PD-1/PD-L1 pathway in neuroinflammation in AD [33, 34]. However, information regarding the expression status of PD-1/PD-L1 on immune cells in AD patients and its correlation with changes in the disease stage is limited. Therefore, we utilized multicolor flow cytometry to analyze T-cell subsets in peripheral blood mononuclear cells (PBMCs) and PD-1/PD-L1 expression in AD patients and healthy volunteers. We aimed to clarify the PD-1/PD-L1 alteration in the etiology/progression of AD patients. Our results show the possibility of using PD-1/PD-L1 as biomarkers and targets in the development of treatment strategies.

## Results

### Alzheimer’s disease patients and healthy volunteers possess similar amounts of peripheral T cells

The pattern of the PBMC population in several neurodegenerative diseases has been demonstrated to significantly differ in several neurodegenerative diseases, such as multiple sclerosis and Parkinson’s disease [35–38]. The variation in PBMC populations indicates either active or suppressive alterations in the immune environment.

Among the PBMC populations, T cells play an essential role in adaptive immunity and can be divided into many subsets by the expression of different surface markers. Changes in the cell number and activity of each T-cell subset are involved in infection, cancer, neurodegenerative disease, and tissue repair [37, 39–41]. Therefore, we attempted to examine whether any T-cell proportion differences exist between patients with Alzheimer’s disease (AD) and healthy volunteers before exploring changes in PD-1 and PD-L1 expression. Among the T-cell subsets, we studied not only subsets of CD4^+^ T cells [typically considered helper T cells (Th)] and CD8^+^ T cells [typically considered cytotoxic T lymphocytes (CTLs)] but also CD4^+^CD25^+^ (IL-2RA) T cells and CD8^+^CD25^+^ (IL-2RA) CTLs. CD25 belongs to the IL-2 receptor alpha chain (IL-2RA), and IL-2 induces T-cell growth and activity. IL-2/IL-2R signaling enhances CD8^+^ CTLs to complete the terminal differentiation of effector cells, stimulates memory cells in a growing state, and helps signal transduction important for the development and survival of regulatory T cells (Tregs) [42]. Once IL-2RA is defective, naïve Tregs cannot maintain immunological homeostasis [43]. This process leads to a rapidly progressive, lethal autoimmune response in murine experiments [44]. Moreover, effector T cells in adaptive immunity include CD8^+^ T cells and natural killer T cells (NKT-like cells, CD3^+^CD56^+^ T cells) [45–47]. CD8^+^ CTLs recognize protein antigens, and NKT cells recognize lipid antigens via nonclassical class I major histocompatibility complex (MHC) molecules (CD1d) [48]. The surface marker of an NKT-like cell, CD56, is a neural cell adhesion molecule (NCAM) that adheres to neurons and glial cells that present the same glycoprotein [49]. Hence, we were interested in the role of NKT-like cells during AD progression.

**Fig. 1:**
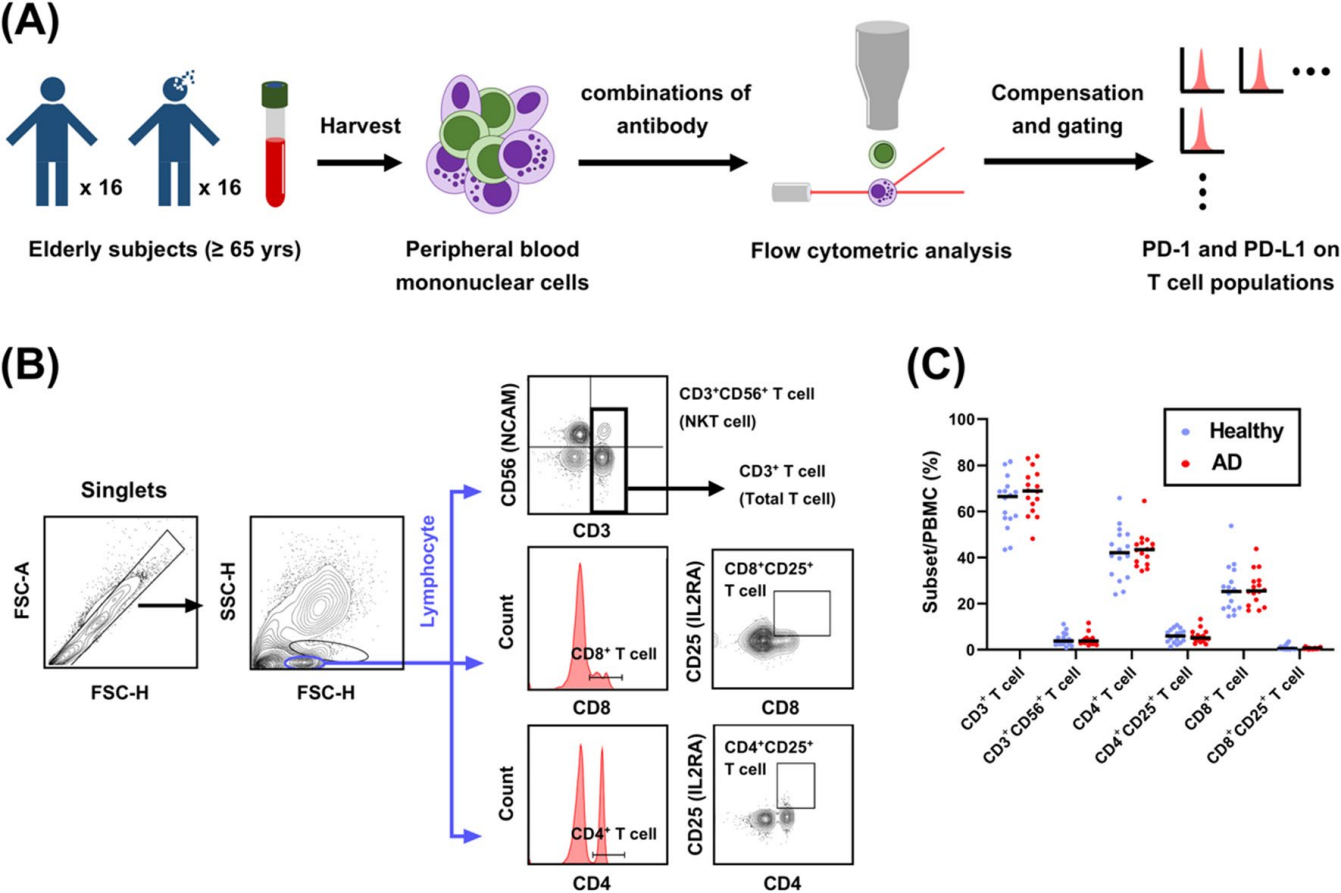
Single-cell-based staining as an indicator in the AD immune status analysis. **A** Experimental and analytical workflow from obtaining PBMCs to the flow cytometric data analysis. Whole blood was collected from 16 healthy volunteers and 16 age-matched AD patients. After purification, PBMCs were bound with antibodies against surface markers and PD-1/PD-L1 before the flow cytometric analysis. The raw data obtained from flow cytometry were compensated to correct the fluorescence spillover, and T-cell subsets were gated by cell surface markers. **B** Gating strategy for different T-cell subsets. Doublet cells were first excluded by the forward scatter height (FSC-H) versus FSC-A density plot. The lymphocyte population was gated by the FSC-H versus side scatter height (SSC-H) plot (indicated by a blue circle), and then, the T-cell subsets were gated by surface markers (CD3, CD4, CD8, and CD56). **C** Comparison of T-cell subset proportions between healthy volunteers and AD patients

We collected blood samples from 16 AD patients and 16 age-matched healthy volunteers (Additional file 1: Figure 1A), lysed erythrocytes, and labeled cell surface molecules. We performed single-cell profiling with flow cytometry as shown in Fig. 1A. The numbers of six T-cell subsets, including CD3^+^ T cells (total T cells), CD8^+^ T cells, CD8^+^CD25^+^ T cells, CD4^+^ T cells, CD4^+^CD25^+^ T cells, and CD3^+^CD56^+^ T cells, were profiled using flow cytometry (Fig. 1B).

The proportions of CD3^+^ T cells, CD3^+^CD56^+^ T cells, CD4^+^ T cells, CD4^+^CD25^+^ T cells, CD8^+^ T cells, and CD8^+^CD25^+^ T cells to the total peripheral T cells did not significantly differ between the AD patients and healthy volunteers (Fig. 1C). Next, the proportions of CD8^+^CD25^+^ T cells to CD8^+^ T cells, CD4^+^CD25^+^ T cells to CD4^+^ T cells, CD8^+^ CTL to CD4^+^ Th cells, and NKT-like cells to CD4^+^ Th cells did not significantly differ between the AD patients and healthy volunteers (Table 1).

**Table 1:** Comparison of peripheral T cell subset ratio between AD patients and healthy volunteers

Population Ratio	Healthy volunteers (n = 16)	Total AD (n = 16)	Exact Sig. (2-sided test)
CD8^+^CD25^+^ (IL-2RA) / CD8^+^	0.036 (0.004–0.102)	0.024 (0.006–0.073)	0.445
CD4^+^CD25^+^ (IL-2RA) / CD4^+^	0.141 (0.050–0.270)	0.134 (0.054–0.286)	0.956
CD4^+^ / CD8^+^	1.914 (0.467–4.400)	1.775 (0.977–3.783)	0.809
CD4^+^ / CD3^+^CD56^+^	2.102 (0.140–7.761)	1.579 (0.279–3.053)	0.724

Mann–Whitney *U* test was used to compare healthy volunteers (n = 16) and AD patients (n = 16)

### PD-L1/2 expression levels in T-cell subsets are upregulated in AD patients

The PD-1/PD-L1 pathway, an adaptive immune-suppressive signaling axis, has been comprehensively studied. Previous cancer- and AD-related research mainly focused on T-cell-expressed PD-1 and tumor cell/APC-expressed PD-L1 [33, 50, 51]. Recently, Diskin et al. uncovered the consequences of PD-L1 ligation in T cells. PD-L1^+^ T cells involve suppressive back-signaling that prevents CD4^+^ T-cell activation and reduces Th1 polarization [52]. Thus, we examined the expression levels of both PD-1 and PD-L1 in T-cell subsets in AD patients and an age-matched normal cohort.

**Fig. 2:**
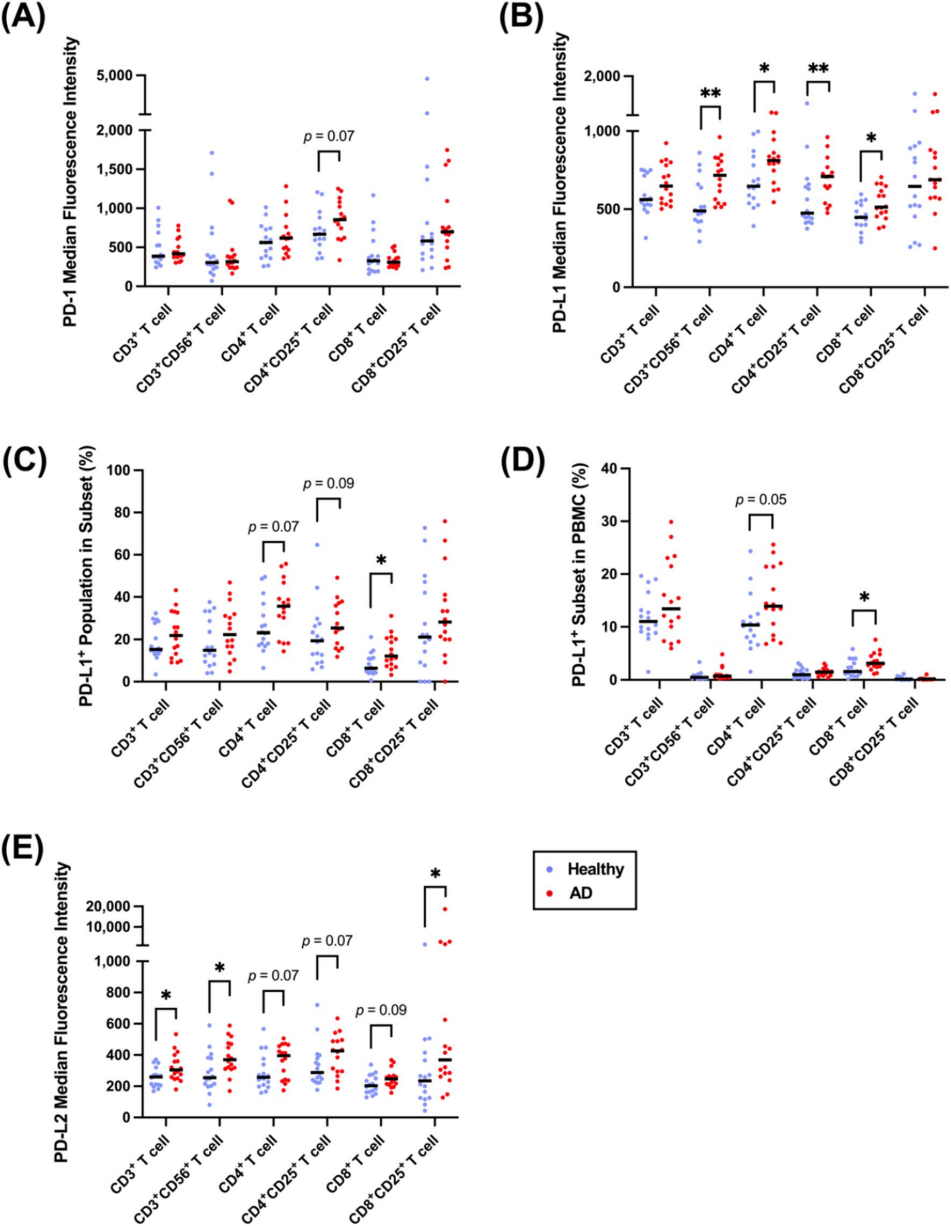
Elevated PD-L1 was observed in AD CD8^+^ T cells and other T-cell subsets. **A**, **B** The median fluorescence intensity of PD-1 and PD-L1 expressed on T-cell subsets in healthy volunteers and AD patients. **C**, **D** The percentage of the PD-L1^**+**^ population in its subset and PBMCs, respectively, in healthy volunteers and AD patients. **E** The median fluorescence intensity of PD-L2 expressed on T-cell subsets in healthy volunteers and AD patients. A Mann‒Whitney *U* test was used to compare the healthy volunteers (n = 16) and AD patients (n = 16); median values are indicated by thick black lines in the scatter plots. *p < 0.05, **p < 0.01

Regarding PD-1, the expression level of PD-1 and the percentage of the PD-1^+^ population in the AD patients’ T-cell subsets (CD3^+^, CD3^+^CD56^+^, CD4^+^, CD8^+^, and CD8^+^CD25^+^ T cells) were similar to those in the healthy volunteers, and only a higher PD-1 expression trend was found in CD4^+^CD25^+^ T cells (Fig. 2A, Additional file 1: Figure 2). In contrast, the expression level of PD-L1 on CD8^+^ T cells and the PD-L1^+^CD8^+^ T-cell number were elevated (Fig. 2B–D). In addition to CD8^+^ T cells, the upregulation of PD-L1 expression on the cell surface was observed in CD3^+^CD56^+^ (NCAM) T cells, CD4^+^ T cells, and CD4^+^CD25^+^ (IL-2RA) T cells in the AD patients (Fig. 2B). Moreover, there was a trend of an enlarged proportion of the PD-L1^+^ subset in the AD patients’ CD4^+^ T cells (Fig. 2C, D). Then, we examined the expression of PD-L2 on T cells since PD-L2 is also a ligand of PD-1 that drives the activation of the PD-1 pathway to inhibit T cells [53]. Significant upregulation of PD-L2 expression on CD3^+^ T cells, CD3^+^CD56^+^ (NCAM) T cells, and CD8^+^CD25^+^ (IL-2RA) T cells was noted in the AD patients (Fig. 2E). Trends of increasing PD-L2 expression on CD4^+^ T cells, CD4^+^CD25^+^ T cells, and CD8^+^ T cells were also noted (Fig. 2E). However, the proportions of the PD-L2^+^ subset in T cells were similar between the AD patients and healthy volunteers (Additional file 1: Figure 3A, B). In summary, a higher expression of the PD-1 ligands PD-L1 and PD-L2 was found in AD patients’ T-cell populations.

### Higher PD-L1 expression levels were found in CD4^+^ and CD8^+^ T cells in moderate AD patients

**Fig. 3:**
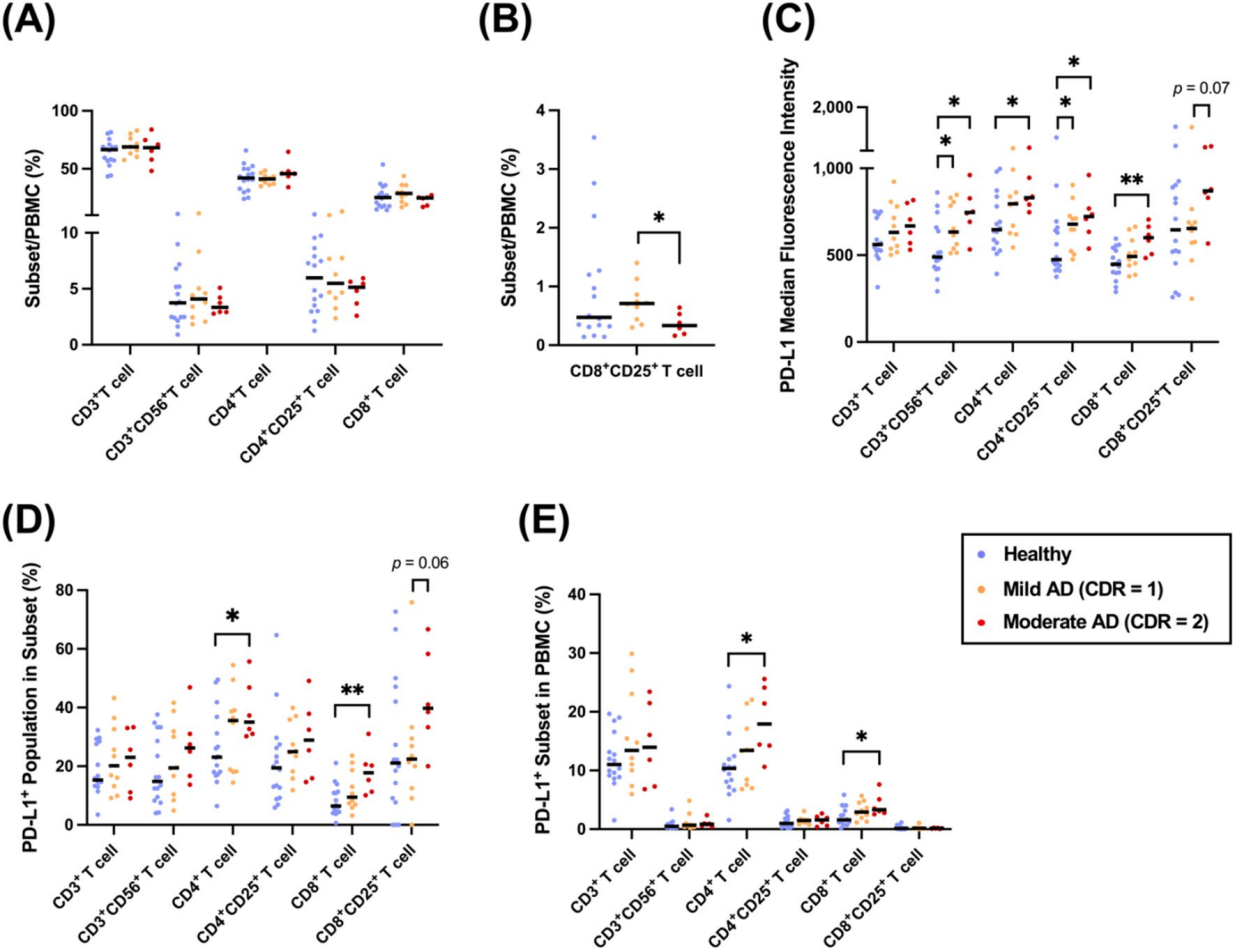
Upregulation of the T-cell PD-L1 immune checkpoint status was detected in the moderate stage of AD. **A** Comparison of T-cell subset proportions among healthy volunteers, mild AD patients, and moderate AD patients. **B** Comparison of CD8^**+**^CD25^**+**^ T-cell population among healthy volunteers, mild AD patients, and moderate AD patients. **C** The median fluorescence intensity of PD-L1 expressed on T-cell subsets in healthy volunteers, mild AD patients, and moderate AD patients. **D**, **E** The percentage of the PD-L1^+^ population in its subset and PBMCs in healthy volunteers, mild AD patients, and moderate AD patients. A Mann‒Whitney *U* test was used to compare healthy volunteers (n = 16), mild AD patients (n = 10), and moderate AD patients (n = 6); median values are indicated by thick black lines in the scatter plots. *p < 0.05, **p < 0.01

The pathological progression of AD can be categorized into three stages based on the CDR as follows: mild, moderate, and severe. Numerical alteration and different activities of CD4^+^ T cells, CD8^+^ T cells, Tregs, and monocytes have been linked to AD progression [14, 54, 55]. The activities of immune cells are modulated by PD-1/PD-L1 expression to combat infection and cancer [56–58]. Based on the results mentioned above, we hypothesized that the number of immune cells and the expression levels of PD-1/PD-L1 might be altered during AD progression. Therefore, we compared the number of T-cell subsets and the expression of PD-1/PD-L1/PD-L2 in patients with mild AD (CDR = 1, age range: 71–88 years) and age-matched patients with moderate AD (CDR = 2, age range: 69–88 years) (Fig. 3A-C, Table 2, Additional file 1: Figure 1B).

**Table 2:** Characteristics of AD patients and healthy volunteers

Variable	Healthy volunteers (n = 16)	Total AD (n = 16)	Mild AD (n = 10)	Moderate AD (n = 6)
Age - years (mean ± SD)	74.9 ± 8.51	80.1 ± 5.77	80.6 ± 5.45	78.5 ± 6.44
Gender—no. (%)				
Female	9 (56.25%)	11 (68.75%)	8 (80%)	3 (50%)
Male	7 (43.75%)	5 (31.25%)	2 (20%)	3 (50%)
Education duration—years	–	4.35 ± 4.12	3.2 ± 3.88	6 ± 4.56
CDR	–	1.375 ± 0.50	1	2
CDR-SOB	–	9.219 ± 3.69	6.85 ± 1.81	13.17 ± 2.23
MMSE	–	11.5 ± 5.47	14.2 ± 3.71	7.00 ± 5.10
MMSE-minus	–	10.125 ± 6.78	5.8 ± 2.57	17.33 ± 5.13

*AD* Alzheimer’s disease, *SD* standard deviation, *CDR* Clinical Dementia Rating, *CDR-SOB* Clinical Dementia Rating Scale Sum of Boxes, *MMSE* Mini-Mental State Examination, *MMSE-minus* Education-based MMSE cut-off value minus MMSE score

We observed that the percentage of CD8^+^CD25^+^ T cells was decreased in the patients with moderate AD compared with that in the mild AD patients (Fig. 3B), suggesting defective terminal differentiation of effector T cells. While examining the PD-1/PD-L1 expression pattern across six T-cell subsets, only CD4^+^CD25^+^ (IL-2RA) PD-1 in the moderate AD patients was significantly higher than that in the healthy group (Additional file 1: Figure 4). In the analysis of PD-1 ligands, we found that the PD-L1 expression levels trended toward a high expression with AD progression. In particular, PD-L1 on CD3^+^CD56^+^ T cells and CD4^+^CD25^+^ T cells was significantly upregulated in the mild AD patients compared with that in the healthy volunteers, while PD-L1 on CD4^+^ and CD8^+^ T cells was upregulated in the moderate AD patients (Fig. 3C). Moreover, PD-L1^+^CD4^+^/CD8^+^ T cells in the moderate AD patients were significantly higher than those in the healthy volunteers (Fig. 3D, E). These results indicate that the mechanisms of inducible PD-L1 expression vary across different T-cell subsets, and PD-L1-inducing factors (e.g., inflammatory molecules and metabolites) were altered in different AD stages [59, 60]. Additionally, PD-L2 on T-cell surfaces also trended toward a high expression during the AD stage (Additional file 1: Figure 5).

### Cognitive impairment and functional decline were related to diminished CD8^+^ T-cells and immune checkpoint status changes

**Fig. 4:**
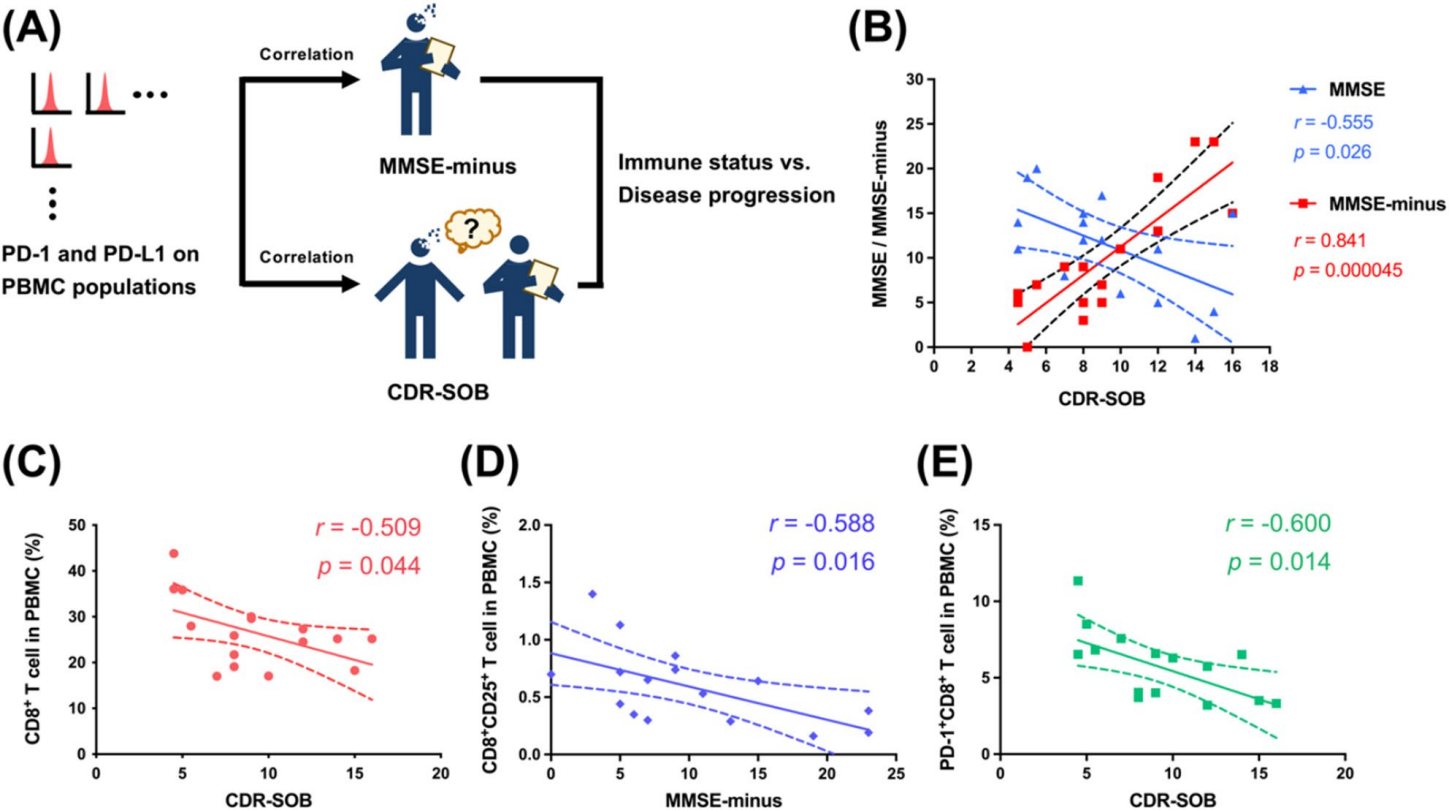
Cognitive and functional declines were associated with diminished CD8^+^ T-cells and status changes in CD25 (IL-2RA) and PD-1. **A** Following the flow cytometric analysis, the PD-1^+^/PD-L1^+^ T-cell subset amounts were correlated with MMSE-minus and CDR-SOB to investigate the relationship between the immune status and disease progression. **B** Pearson correlation between CDR-SOB and MMSE/MMSE-minus (Pearson correlation coefficient *r* = − 0.555, *p* = 0.026 and *r* = 0.841, *p* = 0.000045). **C**–**E** Pearson correlation between CD8^+^ T-cell percentage and CDR-SOB (*r* = − 0.509, *p* = 0.044), between CD8^+^CD25^+^ T-cell percentage and MMSE-minus (*r* = − 0.588, *p* = 0.016), and between PD-1^+^CD8^+^ T-cell percentage and CDR-SOB (*r* = − 0.600, *p* = 0.014)

Blocking the interaction between PD-1/PD-L1 by monoclonal antibodies reinstates impaired T-cell function and is broadly utilized in the treatment of patients with metastatic or late-stage cancer. Several studies have demonstrated that the blockade of the PD-1/PD-L1 pathway in Aβ or tauopathy murine models mitigates cognitive deficits and reduces inflammatory cytokines in the brain [31, 32]. In contrast, Lin et al. found that an anti-PD-1 monoclonal antibody failed to reduce tau pathology or affect cognitive performance in another murine model [61]. Importantly, the correlation between the PD-1/PD-L1 expression status and cognitive impairment needs to be clarified. Hence, we sought to investigate the correlation between AD patients’ PD-1/PD-L1 expression levels and the following cognitive and functional assessment indicators: Clinical Dementia Rating Scale Sum of Boxes (CDR-SOB) and Mini-Mental State Examination (MMSE)-minus (Fig. 4A). MMSE-minus is a newly defined parameter representing “Education-based MMSE cutoff value minus AD patient’s MMSE score” (see “Materials and Methods” section) because the different educational levels of the AD patients significantly biased the evaluation of their cognitive states. MMSE-minus was highly correlated with CDR-SOB (*r* = 0.841, *p* = 0.000045 by Pearson correlation) (Fig. 4B).

The Pearson correlation analysis revealed a negative correlation in the AD patients between CD8^+^ T cells and CDR-SOB (*r* = − 0.509, *p* = 0.044) (Fig. 4C). A reduction in CD8^+^ T cells is related to T-cell exhaustion during chronic viral infections, such as human immunodeficiency virus (HIV), hepatitis B virus (HBV), and the recent outbreak of severe acute respiratory syndrome coronavirus 2 (SARS-CoV-2) [62, 63]. CD8^+^ T cells decline with cognitive impairment, implying that CD8^+^ T cells might be overstimulated, thereby reaching an exhausted state with dysfunctional metabolism and a related reduction in pivotal cytotoxic functions [64]. Moreover, CD8^+^CD25^+^ (IL-2RA) T cells in PBMCs were negatively correlated with the MMSE-minus scores (*r* = − 0.588, *p* = 0.016) (Fig. 4D). Decreased IL-2RA^+^ CTLs might imply a weaker ability for cell differentiation and transformation into memory cells. Additionally, the proportion of PD-1^+^CD8^+^ T cells in PBMCs was negatively correlated with CDR-SOB (*r* = − 0.600, *p* = 0.014) (Fig. 4E).

### CD8^+^ T-cell phenotypic and functional characteristics change across different AD stages

Due to this study lacking cell surface markers, we cannot distinguish different CD8^+^ T-cell (CTL) subsets. Furthermore, the commonly used flow cytometric indicators (median/mean fluorescence intensity and positive population) cannot thoroughly elucidate the PD-1 status in CD8^+^ T-cell subsets in our study. Notably, CD8^+^ T-cell subsets share mutual flow cytometric parameters, including cell size, granularity, and CD8 expression. The data points lie in a high-dimensional space because of the multiple parameters; therefore, the data are challenging to analyze and visualize. To overcome the limitations of high-dimensional data, we adopted an unsupervised dimensionality reduction algorithm, i.e., t-distributed stochastic neighboring embedding (t-SNE).

The t-SNE algorithm maps high-dimensional data points to a low-dimensional space; data points with similar characteristics are clustered on a two-dimensional (2D) plane with a high probability [65]. We gated all subjects’ (healthy volunteers, n = 16; mild AD, n = 10; moderate AD, n = 6) CD8^+^ CTLs with t-SNE as follows:

1. Gather a series of information on phenotypic and functional features, including cell size, granularity, CD8 expression, and PD-1 or PD-L1 expression on each cell from all volunteers’ CD8^+^ CTLs.
2. Cluster CD8^+^ CTLs with similar features.
3. Separate CD8^+^ CTL clusters with different phenotypic features.
4. Visualize CD8^+^ CTL clusters with different densities on a two-axis panel (t-SNE1 and t-SNE2).

**Fig. 5:**
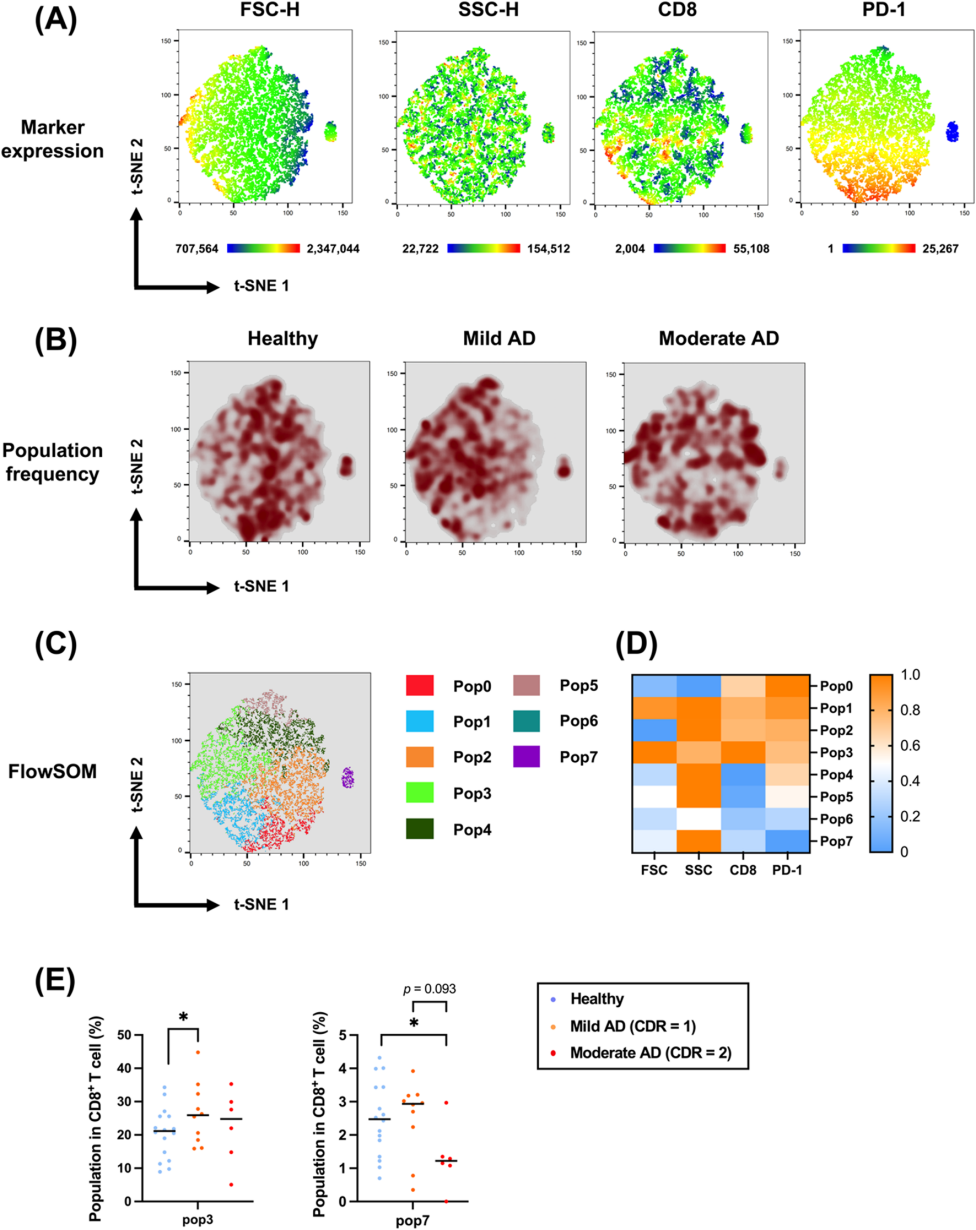
t-SNE analysis of the CD8^+^ T-cell subset with PD-1. **A** t-SNE maps of all subjects demonstrating the numerical values of FSC-H, SSC-H, CD8, and PD-1 expression levels. **B** Pseudocolor smooth density plots displaying the change in the CD8^**+**^ T-cell population frequency with a selected marker, PD-1, among healthy volunteers, mild AD patients, and moderate AD patients. **C** Eight populations on the t-SNE map were clustered by the FlowSOM algorithm. **D** The heatmap demonstrates the frequency of each population with different markers (FSC, SSC, CD8, and PD-1). **E** Populations 3 and 7 in CD8^**+**^ T cells among healthy volunteers, mild AD patients, and moderate AD patients are shown in the scatter plots. A Mann‒Whitney *U* test was used to compare healthy volunteers (n = 16), mild AD patients (n = 10), and moderate AD patients (n = 6); median values are indicated by thick black lines in the scatter plots. *p < 0.05

Therefore, we can distinguish and visualize the complex CD8^+^ T-cell subsets with the following various variables: FSC (cell size), SSC (granularity), CD8 expression, and PD-1/PD-L1 expression. As shown in Fig. 5A, numerous discrete clusters were distributed differently in the pseudocolor density plots among the three groups (healthy volunteers, patients with mild AD, and patients with moderate AD), corresponding to unique cell populations. In mild AD, the patients’ CTLs had a larger size, fewer PD-1^high^-expressing CTLs, and more CD8^high^ CTLs (Fig. 5A, B).

Furthermore, to discover the critical subpopulation changes and obtain quantitative results, we applied the FlowSOM algorithm to sort all subjects’ CD8^+^ CTLs. This machine learning algorithm divided all CD8^+^ CTLs into 8 groups (Pop0-7), and each group had its own characteristic features in terms of the cellular size, granularity, CD8 expression, and PD-1/PD-L1 expression (Fig. 5C, D). Hereafter, the ratios of CD8^+^ T cells in each population in the healthy volunteers, mild AD patients, and moderate AD patients were collected and analyzed. Among the eight populations, the CTLs in Pop3 had a higher CD8 expression and a larger cellular size, and the CTLs were significantly increased in the patients with mild AD compared with those in the healthy volunteers (Fig. 5E; Table 3). The CTLs in Pop7, which had a lower PD-1 expression, were lower in the patients with moderate AD than those in the healthy volunteers. These findings suggest that CTLs tended to have an enlarged cell size and upregulated CD8 expression in the patients with mild AD, while the moderate AD patients had fewer PD-1^negative^ CTL.

**Table 3:** CD8^+^ T cell FlowSOM (w/ PD-1) cluster proportions in healthy volunteers, mild AD, and moderate AD patients

	Median value			Independent-Samples Mann–Whitney *U* Test Significance		
Subject	Healthy volunteer	Mild AD	Moderate AD	Hv vs. Mild	Mild vs. Moderate	Hv vs. Moderate
Pop0 (%)	12.1	5.39	10.495	0.165	0.263	0.693
Pop1 (%)	11.3	15.45	16.35	0.363	0.875	0.693
Pop2 (%)	23.35	22.55	23.6	0.856	0.875	0.914
Pop3 (%)	18.85	25.9	24.8	0.047*	0.713	0.329
Pop4 (%)	21.1	17.05	17.7	0.241	0.562	0.494
Pop5 (%)	4.165	6.235	3.48	0.586	0.118	0.294
Pop6 (%)	0.515	0.58	0.24	0.551	0.313	0.154
Pop7 (%)	2.505	2.935	1.22	0.897	0.093	0.04*

*AD* Alzheimer’s disease, *Pop* FlowSOM population, *Hv* Healthy volunteers

*p < 0.05

**Fig. 6:**
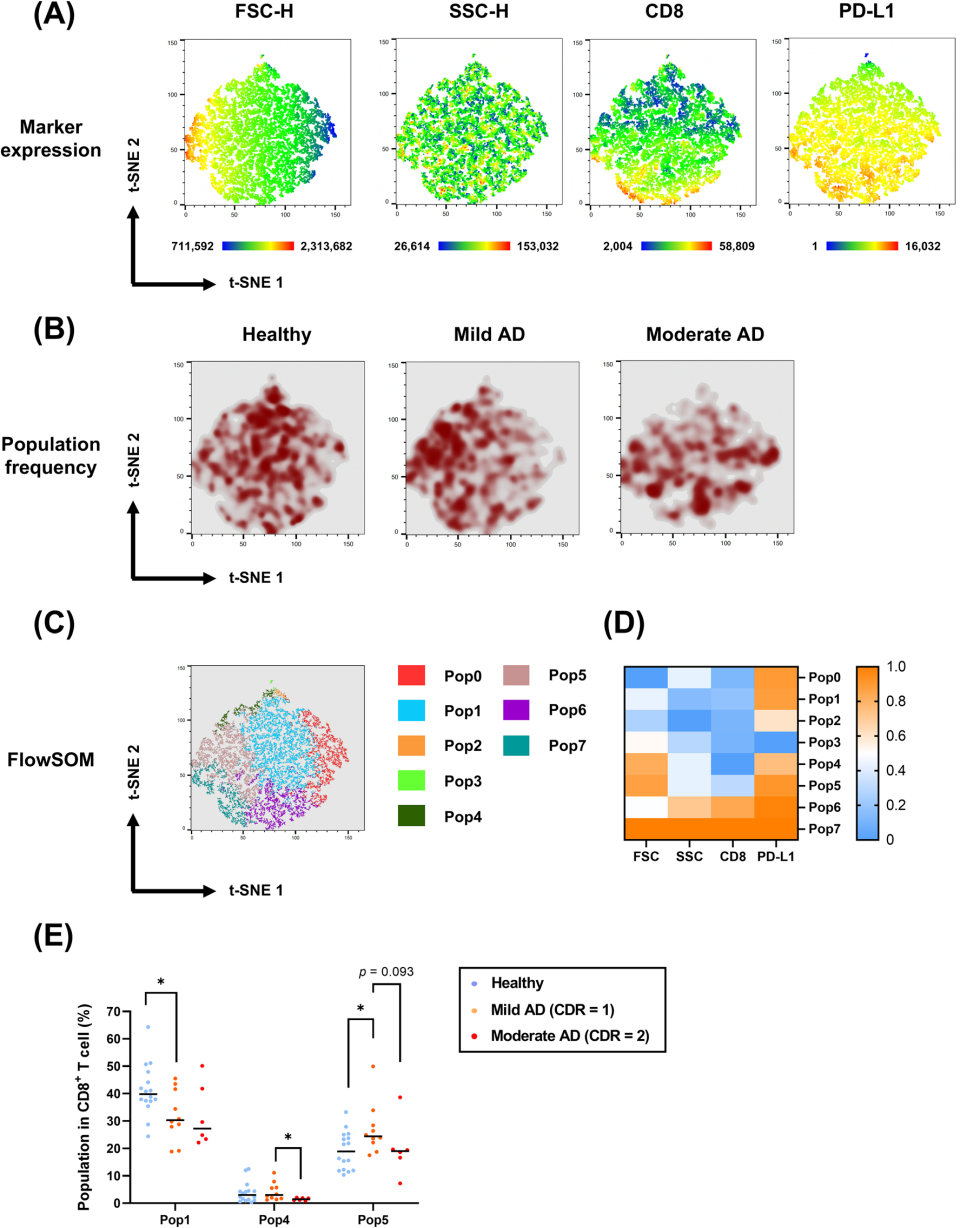
t-SNE analysis of the CD8^+^ T-cell subset with PD-L1. **A** t-SNE maps of all subjects demonstrating the numerical values of FSC-H, SSC-H, CD8, and PD-L1 expression levels. **B** Pseudocolor smooth density plots displaying the change in the CD8^+^ T-cell population frequency with a selected marker, PD-L1, among healthy volunteers, mild AD patients, and moderate AD patients. **C** Eight populations on the t-SNE map were clustered by the FlowSOM algorithm. **D** The heatmap demonstrates the frequency of each population with different markers (FSC, SSC, CD8, and PD-L1). **E** Populations 1, 4, and 5 in CD8^+^ T cells among healthy volunteers, mild AD patients, and moderate AD patients are shown in the scatter plots. A Mann‒Whitney *U* test was used to compare healthy volunteers (n = 16), mild AD patients (n = 10), and moderate AD patients (n = 6); median values are indicated by thick black lines in the scatter plots. *p < 0.05

In the previous section, we found a significant difference in the PD-L1 expression levels between the healthy volunteers and AD patients. We intended to further investigate the possible changes in the CD8^+^ T-cell subsets that express PD-L1 with the t-SNE algorithm. The results revealed that the mild AD patients’ CTLs were larger in cell size, and the moderate AD patients had fewer CD8^low^ CTL populations and PD-L1^low^-expressing CTLs (Fig. 6A, B).

Similarly, we gated eight populations that exhibited noticeable density differences by FlowSOM (Fig. 6C). Among the eight populations, we identified three populations with significant differences among the cohorts. Compared with the healthy volunteers, the mild AD patients’ CTLs from Pop1 (smaller cell) were significantly reduced, while the larger ones (Pop5) were increased. Pop4 (with the lowest CD8 expression) was decreased in the moderate AD patients compared with that in the mild AD patients (Fig. 6E; Table 4). Altogether, these results provide evidence that PD-1/PD-L1-expressing CD8^+^ T-cell subsets are altered with AD progression.

**Table 4:** CD8^+^ T cell FlowSOM (w/ PD-L1) cluster proportions in healthy volunteers, mild AD, and moderate AD patients

	Median value			Independent-Samples Mann–Whitney *U* Test Significance		
Subject	Healthy volunteer	Mild AD	Moderate AD	Hv vs. Mild	Mild vs. Moderate	Hv vs. Moderate
Pop0 (%)	10.15	8.065	16.8	0.551	0.073	0.294
Pop1 (%)	39.8	30.25	27.2	0.047*	0.875	0.115
Pop2 (%)	0.59	0.415	0.135	0.336	0.263	0.07
Pop3 (%)	0.375	0.24	0.2	0.4894	0.635	0.367
Pop4 (%)	2.98	2.95	1.435	0.586	0.031*	0.154
Pop5 (%)	18.9	24.4	19	0.027*	0.093	1
Pop6 (%)	12.9	14.15	15.3	1	0.368	0.541
Pop7 (%)	6.795	12.6	14.25	0.087	0.792	0.115

*AD* Alzheimer’s disease, *Pop* FlowSOM population, *Hv* Healthy volunteers

*p < 0.05

## Discussion

### Alteration in the PD-L1/L2-PD-1 status in AD patients’ peripheral T cells

Most AD patients are elderly and vulnerable to various infections and inflammation. Each peripheral blood infection and inflammation might lead to a transient or long-term worsening of cognitive changes. Our work contributes to previous publications that tested whether PBMCs might provide insight into the pathogenesis of AD [66–69]. To verify the relationship between the peripheral T-cell immunological status and cognitive fluctuation in AD patients, we examined T-cell immunological changes between healthy volunteers and patients with different AD stages.

We observed an upregulation of PD-L1/PD-L2 on several T-cell subsets in the AD patients, particularly CD8^+^ T cells and CD4^+^ T cells. In contrast, there was no significant difference in the PD-1 expression level. Relatively few studies have explored T-cell-expressing PD-L1. Notably, PD-L1^+^ T cells restrain effector T cells and suppress neighboring PD-1^+^ T cells in the tumor microenvironment [52], implicating PD-L1-mediated immune suppression. In addition, the upregulation of PD-L1 in immune cells is dependent on either TLR- or IFN-γ-mediated signaling pathways [70] or extrinsically controlled by the inflammatory cytokines IL-6 and IL-10 in a MAPK/cytokine/STAT-3-dependent manner [71]. We postulated that the upregulation of PD-L1 might be induced by inflammation and ultimately result in the formation of an immunosuppressive-prone environment in AD patients. This postulation can be further supported by demolishing different inflammatory signaling pathways in AD murine models, examining PD-L1 expression, and monitoring AD development. In summary, we revealed peripheral PD-L1/PD-L2 inhibitory stress from CD4^+^ and CD8^+^ T cells in AD patients, leading to a diminished immune response.

Our study also uncovered an upregulation of PD-L1 expression with disease progression. PD-L1 can be induced by inflammatory molecules and intracellular metabolites [59, 71]. Such alteration in the PD-L1 expression status revealed that inducible PD-L1 expression varies across different AD stages probably resulting from divergent sensitivities to PD-L1-inducing factors. Moreover, CD8^+^ T cells and CD8^+^CD25^+^ (IL-2RA) T cells decreased with progression, possibly resulting from T-cell exhaustion, which causes or affects AD deterioration. AD patients are infected by various viruses and/or bacteria during aging. Each infection episode exhausts the CD8^+^ T cells of AD patients, which correlates with cognitive decline. The above findings might also suggest a possible mechanism in delirium, which is common in elderly and demented patients during acute inflammation and infection. CD8^+^ T-cell exhaustion, in addition to the previous neurotransmitter dysregulation hypothesis, could provide newer insight into the mechanism of delirium in elderly individuals [72].

We additionally implemented the t-SNE algorithm and identified, to the best of our knowledge, for the first time that PD-1 expression is reshaped in the CD8^+^ T-cell subset with AD progression, which was not discovered using a conventional flow cytometric analysis [33]. With this unprecedented observation, we speculate that CD8^+^ T cells are involved in the late stage of T-cell activation since PD-1 acts as a senescence marker of T-cell differentiation and is prone to be inhibited by the PD-1/PD-L1 pathway in moderate AD patients. It will be necessary for future work to further verify this hypothesis by observing the T-cell activation status (e.g., CD69 expression) after treatment with an anti-PD-1 monoclonal antibody in AD patients and comparing it with cognitive changes. In addition, our t-SNE computational results might serve as preliminary references for PD-1/PD-L1 blockade therapies for the treatment of patients at different AD stages. Moreover, an upregulation of CD8 expression and a larger cell size were found in the mild AD stage. CD8 expressed on T cells acts as a coreceptor for the T-cell receptor (TCR), which is required for a stable complex between the TCR and MHC class I, enhancing peptide sensitivity by one million-fold or more [73]. Depleting CD8 expression leads to reduced CD8^+^ T-cell sensitivity for antigens and the inefficiency of antigen-specific cytotoxicity [74]. In addition, a previous study revealed that activated CD8^+^ T cells have a significantly larger diameter than resting cells [75]. These features imply that the cytotoxic capability of CD8^+^ T cells might be active in patients with mild AD.

The T-cell-expressing PD-1/PD-L1 reshaping observed in this study suggests a negative immune regulation status in AD progression. This status change in AD patients might indicate concurrent inflammation states, such as viral/bacterial infections, postinfectious inflammation, and probable metabolic syndrome. Mediation of the PD-1/PD-L1 pathway might restore T-cell activity, thereby triggering immune responses and alleviating AD pathology.

### The immune system could act as a friend, a foe, or both in AD

Cytotoxic T cells have been delineated as participants in developing neurodegenerative diseases, such as Parkinson’s disease and multiple sclerosis [38, 76, 77]. The activation of antigen-specific cytotoxic T-cell lineages might be induced by certain infections, resulting in a probable T-cell-dominated attack on neuron cells with similar antigens. Our results demonstrate the downregulation of PD-1^negative^ CD8^+^ T cells and the numerical decline in CD8^+^ T cells with AD progression. Here, we propose that the cytotoxicity of CD8^+^ T cells destroys neuronal cells in AD, thereby exacerbating the disease. In early-stage AD, peripheral immunity is shaped to an immunosuppressive-like environment by upregulating PD-L1 on immune cells. However, the downregulation of PD-1 on CD8^+^ T cells enables the evasion of PD-L1 inhibition, thereby attacking neurons. Similarly, the activation of specific T cells was related to the mild disease stage in AD patients in a previous study [14]. In late-stage T-cell activation, the number of CD8^+^ T cells declines due to exhaustion. Concurrently, the negative feedback in patients’ peripheral blood suppresses CD8^+^ T cells with high PD-L1 expression and restores PD-1 expression in CD8^+^ T cells. Despite T-cell suppression, the CNS is damaged, leading to cognitive decline/impairment. The proposed model can be preliminarily validated by identifying the clonal expansion of AD-associated antigen-specific T cells and examining the cytotoxicity mediated by the antigen expressed on neuron cells. Following the preceding validation, we can further confirm this model by applying anti-CD3 antibodies in an AD murine model, which selectively depletes pathogenic T cells while preserving regulatory T cells [78] and observing cognitive restoration.

In contrast, our findings provide another AD pathogenesis model, i.e., dysfunctional immunity cannot preserve neuroregeneration. Neurons undergo damage for several reasons, including oxidative stress, inflammation [79], mitochondrial dysfunction [80], impaired autophagy–lysosomal activities [81], and the perturbation of vesicle trafficking and synapse dysfunction. Severely damaged neurons are replaced by glial cells [82]. The immune system contributes to neurogenesis by recruiting T cells and activating microglia [83–86]. AD progresses slowly, presenting a long preclinical phase, with the initial deposition of AD pathology estimated to begin approximately 10–15 years before the onset of clinical symptoms [87, 88]. Specific CD8^+^ T-cell subsets might maintain such a steady state. Nevertheless, with the diminished number of CD8^+^ T cells and the upregulation of PD-L1, the peripheral blood reshapes into an immunocompromised environment and causes neurogenesis malfunction. With disease progression, CD8^+^CD25^+^ (IL-2RA) T cells undergo exhaustion accompanied by increased PD-L1-expressing CD4^+^ or CD8^+^ T cells. Without normal neurogenesis, damaged neurons exhibit an aberrant transmission of electrical signals [89, 90] and cause neurological disorders. Rosenzweig et al. blocked the PD-1/PD-L1 pathway before cognitive deficits and observed cognitive recovery in a tauopathy murine model [31], which supports this model. The following three distinctive approaches can preliminarily verify this putative model: (1) observing AD development in different T-cell-deficient murine models; (2) applying anti-PD-1 or anti-PD-L1 monoclonal antibodies in AD patients and correlating PD-1/PD-L1 blockade with damaged brain repair; and (3) observing whether the regulation of T-cell activity can alter the rate of deterioration and cognitive impairment.

## Limitations

From the perspective of practical neurology, we enrolled outpatients who met the clinical NIA-AA 2011 criteria/DSM-5 criteria for probable Alzheimer’s disease with a typical clinical course, brain MRI findings, and neurocognitive tests. However, the lack of potential biomarkers (amyloid/tau) in cerebrospinal fluid (CSF) and blood was our study limitation, especially while we attempted to bridge the clinical setting to basic research.

As a cross-sectional observational study, this study precludes causal inference, which is required to strengthen the connection between our data and clinical practice. To mitigate this limitation, a combination of in vitro and/or in vivo functional assays, medical imaging analyses (e.g., functional magnetic resonance imaging and amyloid positron emission tomography), and medical records could be adopted in further research. Moreover, we had a small sample size and uneven sex distribution due to difficult enrollment. Our study was subject to sampling errors, leading to incorrect data evaluation and interpretation. Despite this limitation, this study revealed significant alterations in the PD-1/PD-L1 expression status in AD patients by utilizing nonparametric statistical methods (Mann‒Whitney *U* test and Kruskal‒Wallis test). Finally, long-term follow-up data would be mandatory to observe temporal relationships.

## Conclusions

We demonstrated an alteration in PD-1/PD-L1 (L2) expression in AD patients’ peripheral T-cell subsets, which changed the regulatory mechanism of immune homeostasis in different disease stages. The upregulation of PD-L1 (L2) expression causes probable immunosuppression in AD. In addition, the CD8^+^ T-cell data from all subjects were redefined with a combination of phenotypic and functional information (cell size, granularity, CD8, and PD-1/PD-L1), and these high-dimensional data points were visualized with the t-SNE algorithm. Our t-SNE analyses found a characteristic change in CD8^+^ T cells at different AD stages and identified a decreased PD-1^negative^ CD8^+^ T-cell population in moderate AD. This finding implies that the sensitivity of T cells to the inhibitory ligand PD-L1 is higher in moderate AD. The findings in our study provide new evidence that the regulation of immune homeostasis is connected to AD and its different stages. Furthermore, PD-1/PD-L1 on peripheral T cells might be a potential factor interacting with AD; it could be rational to consider immune checkpoint blockade as a potential treatment for AD.

## Methods

### Study participants

Peripheral blood samples were collected from 16 AD patient volunteers (age range: 69–88 years, mean ± SD: 80.1 ± 5.77) and 16 age-matched healthy volunteers (age range: 65–91 years, mean ± SD: 74.9 ± 8.51) from the National Taiwan University Hospital Hsin-Chu Branch. This study was approved by the National Taiwan University Hospital Hsin-Chu Branch Institutional Review Board (IRB number: 108-008F) and performed in accordance with all relevant ethical regulations. All participants or their legal representatives provided written informed consent. All AD patients met the criteria for “probable AD” by the National Institute on Aging and Alzheimer’s Association (NIA-AA) and “dementia” criteria by the Diagnostic and Statistical Manual of Mental Disorders, fifth edition (DSM-V). Brain magnetic resonance imaging or computed tomography of the AD patients was reviewed, and patients with destructive lesions, such as brain tumors, cerebral infarcts, cerebral hemorrhage, abnormal thyroid function, vitamin B12 deficiency, and folic acid deficiency, were excluded from this study.

### Cognitive and functional assessment indicators

The Clinical Dementia Rating (CDR) was rated by clinicians to evaluate the AD patients’ cognitive performance and daily function. The CDR global score is a summary of 6 cognitive domains, including memory, orientation, judgment and problem solving, community affairs, home and hobbies, and personal care. Mild AD was defined as CDR = 1, moderate AD was defined as CDR = 2, and severe AD was defined as CDR = 3. To track general changes across different stages of AD, we utilized the Clinical Dementia Rating Scale Sum of Boxes (CDR-SOB). CDR-SOB was derived by adding six CDR domain box scores with a sum minimum of 0 and maximum of 18.

The Mini-Mental State Examination (MMSE) is used worldwide to evaluate cognitive function with a total score of 30. A score less than 23 on the MMSE is considered cognitive impairment. However, the educational level is a significant bias in evaluating AD patients’ cognitive states. In our study, the average years of education was only 4.35 years. The Taiwanese population-based cutoff value score for MMSE by education years was defined as 17 for illiterate, 19 for less than 4 years, 24 for 6–8 years, and 27 for 9–12 years of education [91]. Hence, this study defined a new parameter called “‘MMSE-minus’ to represent “education-based MMSE cutoff value minus AD patient’s MMSE score.” A higher MMSE-minus score indicates worse cognitive performance, while a lower score indicates superior cognitive performance.

### Red blood cell lysis

Whole blood was drawn with a VACUETTE® blood collection tube coated with lithium heparin anticoagulant (Greiner Bio-One, Kremsmünster, Austria). Red blood cells (RBCs) were lysed using RBC lysis buffer (0.15 M NH_4_Cl, 10 mM NaHCO_3_, 1 mM EDTA) at room temperature. After removing the RBCs, peripheral blood mononuclear cells (PBMCs) were harvested, washed, and resuspended in phosphate-buffered saline (PBS).

### Antibodies for immunofluorescent staining

All antibodies used for immunofluorescent staining (BioLegend, San Diego, CA, USA) included fluorescein isothiocyanate (FITC)-conjugated mouse anti-human CD3 (OKT3), CD4 (OKT4), CD8 (HIT8a), phycoerythrin (PE)-conjugated mouse anti-human CD3 (OKT3), CD4 (OKT4), goat anti-mouse IgG Fc (Poly4053), allophycocyanin-conjugated mouse anti-human CD25 (BC96), CD45 (2D1), PE/Cy5-conjugated mouse anti-human CD56 (NCAM) (5.1H11), mouse anti-human PD-L1 (29E.2A3), PD-L2 (24F.10C12), and PD-1 (NAT105).

### Flow cytometry analysis

PBMCs were stained with monoclonal antibodies for membrane labeling and incubated at 4 °C in the dark for an hour. Following staining and centrifugation, the cell pellets were washed, resuspended in PBS, and fixed with 0.5% paraformaldehyde/PBS. PBMCs were acquired on an Accuri C6 Flow Cytometer (Becton Dickinson, Franklin Lakes, NJ, USA) equipped with a diode laser and a solid-state laser operating at 640 nm and 488 nm. The samples were first run using single fluorochrome staining for color compensation. Singlet events were first gated on the forward-scatter area (FSC-A) and forward-scatter height (FSC-H) and then gated on FSC-H and side-scatter height (SSC-H) properties of lymphocytes and monocytes. Lymphocyte populations were gated on CD3, CD4, CD8, CD56, and CD25 expression. FlowJo software (FlowJo, LLC, Becton Dickinson) was used to analyze the flow cytometric results. Since the distributions of the flow cytometric fluorescence intensity of immune checkpoints expressed on PBMCs rarely align with a normal distribution, we utilized the median fluorescence intensity (MFI) in the subsequent analysis.

### t-distributed stochastic embedding (t-SNE) analysis

t-distributed stochastic neighbor embedding (t-SNE) is a machine learning algorithm developed by Laurens van der Maaten and Geoffrey Hinton and is a technique used for dimensionality reduction [65]. The t-SNE algorithm embeds high-dimensional data for visualization in a low-dimensional space, such as a two- or three-dimensional space. The axes of the low-dimensional spaces are given in arbitrary units.

In this study, t-SNE was implemented using the ‘t-SNE’ plugin in FlowJo software. The Flow Cytometry Standard (FCS) 3.0 files from both AD patients and healthy volunteers were imported, concatenated to a single FCS file, and visualized with the t-SNE plugin in FlowJo. The parameters were used at the default values (perplexity = 20, learning rate = 200, iterations = 1000, and theta = 0.5) in all analyses.

## FlowSOM clustering method

FlowSOM is an algorithm used for clustering and visualizing high-dimensional flow cytometry datasets. The FlowSOM algorithm uses a self-organizing map (SOM), an unsupervised technique for clustering and dimensionality reduction [92]. In this study, FlowSOM was implemented using the FlowSOM plugin in FlowJo software. The number of clusters was set as 8, which is the default value in the FlowJo software.

### Statistical analysis

The statistical analyses were performed using SPSS statistical software (SPSS Inc., IBM, Chicago, IL, USA). The immunological difference between the AD patients and healthy volunteers was analyzed with nonparametric tests as follows: an independent-samples Mann‒Whitney *U* test (two categorical variables) and Kruskal–Wallis test (more than two categorical variables) due to small-size clinical data. The correlations between the immunological difference and the clinical neuropsychiatric indicators were analyzed with a Pearson correlation analysis. The normality of the data and autocorrelation in residuals were confirmed by the Shapiro‒Wilk test and detected by the Durbin-Watson test, respectively, before the linear regression analysis. All statistical tests and *p* values are two-tailed. All figures with statistical analyses were generated with GraphPad Prism 9 (GraphPad Software Inc., San Diego, CA, USA).

## Supplementary Information

**Additional file 1: Figure 1**. Age-matched elderly human volunteers. (A) All subjects in this study were age-matched and included 16 healthy volunteers and 16 AD patients. (B) Patients with mild AD (CDR = 1; n = 10) and moderate AD (CDR = 2; n = 6) were age-matched. The data are shown as the mean ± SD. A Mann‒Whitney *U* test was used for the comparison. ns: not significant.** Figure 2**. PD-1-expressing T-cell subsets in healthy volunteers and AD patients. The percentage of the PD-1^+^ population in its subset in healthy volunteers and AD patients. A Mann‒Whitney *U* test was used to compare healthy volunteers (n = 16) and AD patients (n = 16); median values are indicated by thick black lines in the scatter plots.** Figure 3**. PD-L2-expressing T-cell proportions in healthy volunteers and AD patients. (A, B) The percentage of the PD-L2+ population in its subset and PBMCs in healthy volunteers and AD patients. A Mann‒Whitney *U* test was used to compare healthy volunteers (n = 16) and AD patients (n = 16); median values are indicated by thick black lines in the scatter plots.** Figure 4**. PD-1 expression on T-cell subsets in different AD stages. The median fluorescence intensity of PD-1 expressed on T-cell subsets in healthy volunteers, mild AD patients, and moderate AD patients. A Mann‒Whitney *U *test was used to compare healthy volunteers (n = 16), mild AD patients (n = 10) and moderate AD patients (n = 6); median values are indicated by thick black lines in the scatter plots.** Figure 5**. PD-L2 expression on T-cell subsets in different AD stages. The median fluorescence intensity of PD-L2 expressed on T-cell subsets in healthy volunteers, mild AD patients, and moderate AD patients. A Mann‒Whitney *U* test was used to compare healthy volunteers (n = 16), mild AD patients (n = 10) and moderate AD patients (n = 6); median values are indicated by thick black lines in the scatter plots.

## Data Availability

All data generated or analyzed during this study are included in this published article and its additional files.
