# Supplementary material for: A change of PD-1/PD-L1 expression on peripheral T cell subsets correlates with the different stages of Alzheimer's Disease
Source: Cell Biosci. 2022 Sep 30;12:162. doi: 10.1186/s13578-022-00897-1 (PMC9524741; doi:10.1186/s13578-022-00897-1)
Supplement: Supplementary file 1 — Additional file 1: Figure 1. Age-matched elderly human volunteers. (A) All subjects in this study were age-matched and included 16 healthy volunteers and 16 AD patients. (B) Patients with mild AD (CDR = 1; n = 10) and moderate AD (CDR = 2; n = 6) were age-matched. The data are shown as the mean ± SD. A Mann‒Whitney U test was used for the comparison. ns: not significant. Figure 2. PD-1-expressing T-cell subsets in healthy volunteers and AD patients. The percentage of the PD-1+ population in its subset in healthy volunteers and AD patients. A Mann‒Whitney U test was used to compare healthy volunteers (n = 16) and AD patients (n = 16); median values are indicated by thick black lines in the scatter plots. Figure 3. PD-L2-expressing T-cell proportions in healthy volunteers and AD patients. (A, B) The percentage of the PD-L2+ population in its subset and PBMCs in healthy volunteers and AD patients. A Mann‒Whitney U test was used to compare healthy volunteers (n = 16) and AD patients (n = 16); median values are indicated by thick black lines in the scatter plots. Figure 4. PD-1 expression on T-cell subsets in different AD stages. The median fluorescence intensity of PD-1 expressed on T-cell subsets in healthy volunteers, mild AD patients, and moderate AD patients. A Mann‒Whitney U test was used to compare healthy volunteers (n = 16), mild AD patients (n = 10) and moderate AD patients (n = 6); median values are indicated by thick black lines in the scatter plots. Figure 5. PD-L2 expression on T-cell subsets in different AD stages. The median fluorescence intensity of PD-L2 expressed on T-cell subsets in healthy volunteers, mild AD patients, and moderate AD patients. A Mann‒Whitney U test was used to compare healthy volunteers (n = 16), mild AD patients (n = 10) and moderate AD patients (n = 6); median values are indicated by thick black lines in the scatter plots. [file 13578_2022_897_MOESM1_ESM.pdf]

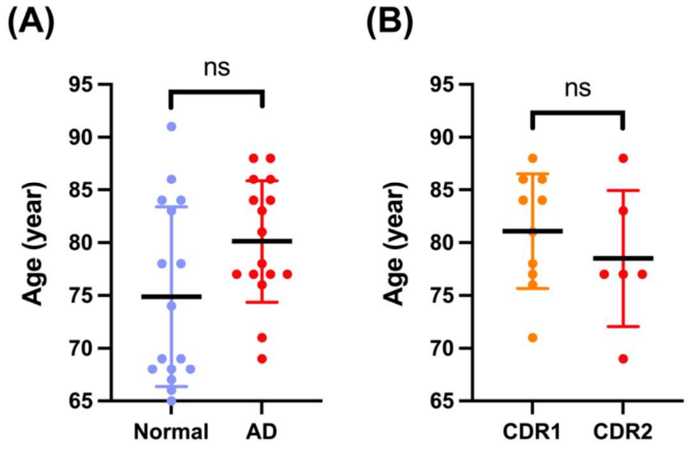

Supplementary Figure 1

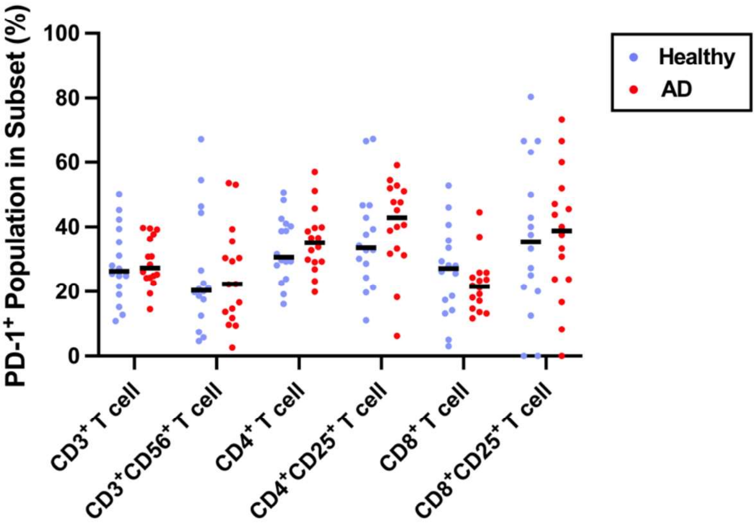

Supplementary Figure 2

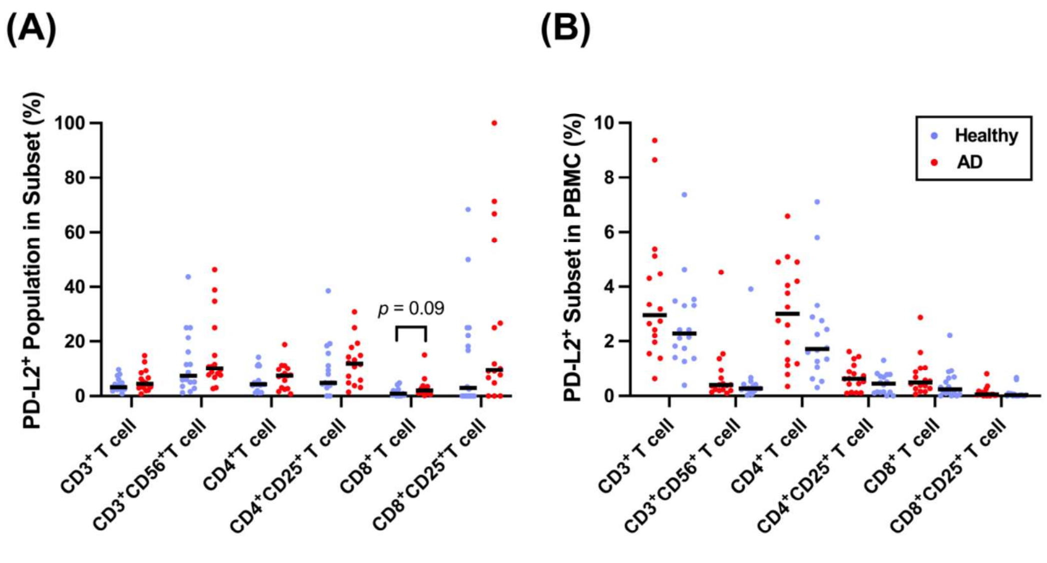

Supplementary Figure 3

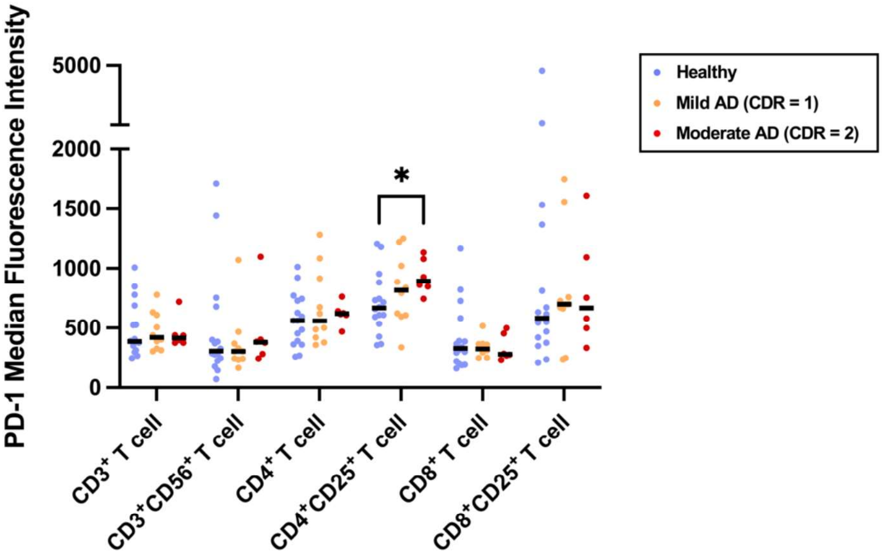

Supplementary Figure 4

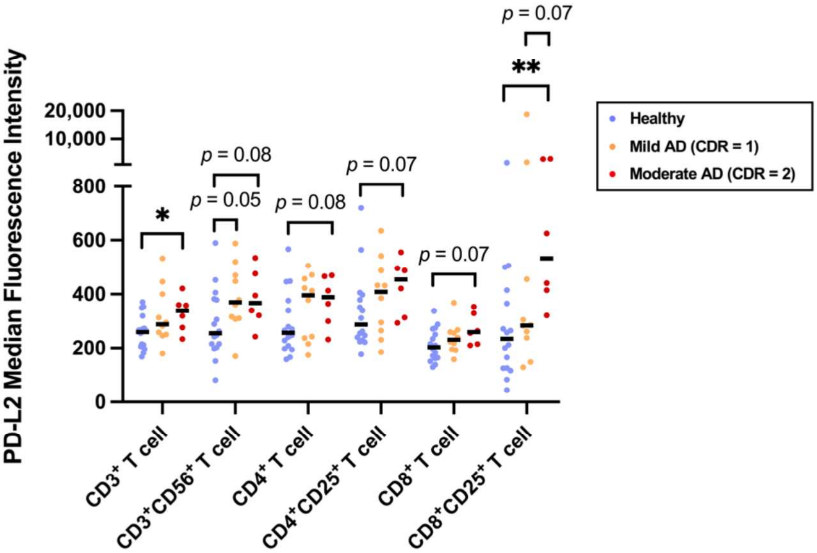

Supplementary Figure 5
